# Clinical Outcomes and Healthcare Resource Utilization of Ceftolozane/Tazobactam in Vulnerable Patient Populations

**DOI:** 10.3390/antibiotics15020179

**Published:** 2026-02-06

**Authors:** Emre Yücel, Alex Soriano, Florian Thalhammer, Stefan Kluge, Mike Allen, Jessica Levy, Huina Yang, Sunny Kaul

**Affiliations:** 1 Merck & Co., Inc., P.O. Box 1000, Rahway, NJ 07065, USA; 2 Hospital Clinic, 08036 Barcelona, Spain; asoriano@clinic.cat; 3 Division of Infectious Diseases and Tropical Medicine, Department of Medicine I, Medizinische Universität Wien, 1090 Wien, Austria; 4 Department of Intensive Care Medicine, University Medical Center Hamburg-Eppendorf, 20251 Hamburg, Germany; 5 MSD (UK) Limited, London, 120 Moorgate, London EC2M 6UR, UK; 6 Tan Tock Seng Hospital, 11 Jln Tan Tock Seng, Singapore 308433, Singapore; 7 Royal Brompton & Harefield NHS Foundation Trust, Britten St, London SW3 6PY, UK

**Keywords:** ceftolozane/tazobactam, sepsis, immunocompromised, transplant, cancer, febrile neutropenia

## Abstract

**Background:** AMR is a public health concern which leads to high global morbidity and mortality. Immunocompromised patients, who are more susceptible to contracting potentially life-threatening infections, are faced with reduced treatment options due to emerging AMR. Ceftolozane/tazobactam is a novel β-lactam/β-lactamase inhibitor which displays effectiveness against resistant Gram-negative infections. **Methods:** SPECTRA was a multinational, observational study conducted in seven countries including 617 patients who received ≥48 h of ceftolozane/tazobactam. Medical-record data were collected up to 6 months before treatment and 30 days after the final dose or until death. This analysis describes clinical outcomes and healthcare resource use in patients with sepsis or who were immunocompromised, specifically in patients with hematologic malignancy with and without solid tumor, febrile neutropenia, and solid organ transplant patients. **Results:** Clinical success ranged from 50.0% in patients with hematologic malignancy and solid tumor to 69.4% in 38 patients with febrile neutropenia. All-cause in-hospital mortality was 23.1–42.9%, with the lowest rates in patients with solid organ transplant. ICU admission was 46.4–68.2% across subpopulations (excluding febrile neutropenia) with the lowest rates in patients with hematologic malignancy. ICU length of stay was lowest within transplant patients (9 days) and highest within the hematologic malignancy and solid tumor population (32 days). **Conclusions:** The results from this sub analysis of SPECTRA showed that ceftolozane/tazobactam was associated with clinical success in the selected immunocompromised and sepsis patient populations and may lead to reduced morbidity, mortality, and healthcare-resource use. Further research is required to standardize treatment protocols and improve patient outcomes.

## 1. Introduction

Infections caused by Gram-negative, antibiotic-resistant (AMR) bacteria are on the rise, leading to increased morbidity and mortality. In 2019, AMR infections were attributed to 1.27 million deaths, causing the World Health Organization (WHO) to declare AMR as a global health concern [1]. These infections lead to increased healthcare resource utilization (HCRU) such as intensive care unit (ICU) admission which can cost up to US $55 billion every year [2]. It is estimated that HCRU costs will increase to a total of US $1 trillion by 2050 [1].

Appropriate antibiotic treatment is crucial for vulnerable populations like those who are immunocompromised, including transplant recipients who have weakened immune systems due to immunosuppressive therapy, leading to a significantly higher risk of serious infections [3]. Immunocompromised individuals often have weakened immune systems or are undergoing treatments that suppress their immune responses, making them more susceptible to opportunistic infections and potentially life-threatening complications [3]. In a 2023 retrospective cohort study of 393 patients, infection-related mortality rate within 7 days was reported to be higher in immunocompromised patients compared to immunocompetent patients (26.1 vs. 13.1%, *p* = 0.002) [4]. Moreover, infection-related death in solid-organ transplant patients is significant, ranging between 13 and 50% [5]. Significant costs are also associated with post-transplantation infections, ranging from US $17,248 to $246,548 across five studies [6].

Sepsis is a condition resulting from dysregulated immune response to infection [7]. Vulnerable populations such as immunocompromised and transplant recipients are at high risk of sepsis, which within these populations is associated with higher risk of severe outcomes and mortality [8]. In 2020, 11 million deaths globally were attributed to sepsis, which accounts for approximately 20% of mortality worldwide [9]. Furthermore, sepsis-related costs are significant and have been estimated at US $32,000 per patient [9], highlighting the important clinical and economic impact associated with the condition. Early recognition and treatment are crucial to improve outcomes for patients with sepsis [7,8]. However, patients are often treated with broad-spectrum antibiotics which contribute to the growing prevalence of AMR, in turn leading to higher morbidity, mortality, and HCRU [7].

Patients with hematologic malignancies (HM) and those undergoing chemotherapy are also at increased risk of infection [10]. In 2020, approximately 700,000 deaths were attributed to hematological cancers globally [11]. Approximately 60% of all deaths in cancer patients with underlying HM are due to infections [12]. Febrile neutropenia (FN), often associated with cancer treatment, increases the risk of serious bacterial infections. FN typically leads to serious complications in 25–30% of cases and to mortality in 9–12% of cases, with the mortality rate reaching up to 50% [13,14].

Vulnerable patient populations such as immunocompromised patients, transplant patients, or patients with cancer, FN, or sepsis are at higher risk of morbidity and mortality due to AMR infections resulting from their compromised health status, and caring for these patients is associated with higher healthcare costs compared to infections in immunocompetent patients [8]. Consequently, there is a need for novel therapeutics against multi-drug resistant (MDR) organisms, particularly for pathogens classified as high priority by the WHO such as *Pseudomonas aeruginosa* [15]. Ceftolozane/tazobactam (C/T) is a β-lactam/β-lactamase inhibitor, consisting of a combination of ceftolozane and tazobactam. It has shown effectiveness against a range of Gram-negative pathogens, including MDR *P. aeruginosa* [16]. C/T is often used in vulnerable populations including immunocompromised individuals, transplant recipients, and those with FN or sepsis, particularly for infections caused by MDR *P. aeruginosa* [17]. C/T has shown to be a valuable option when other treatments are ineffective due to AMR, offering a balance of safety and efficacy.

This manuscript explores clinical outcomes and HCRU associated with C/T use in a number of vulnerable patient populations, including those with sepsis, those who were immunocompromised including cancer patients and transplant recipients, and patients with FN.

## 2. Results

### 2.1. Patient Characteristics

A total of 617 patients were included in the SPECTRA study. Of these, 277 had sepsis, 268 were immunocompromised, 160 were transplant patients, 38 had FN, and 140 had cancer. Patient baseline characteristics can be found in Table 1 with the exception of FN. The mean age of the outlined vulnerable patient populations ranged from 50.3 to 63.8 years, with the majority of patients being male (66.0–75.0%).

Comorbidities and medical history were reported for patients with sepsis and immunocompromised states. Within the sepsis patient population, 20.6% had no comorbidities and 79.4% had at least one comorbidity, with the most common being an immunocompromised state and heart disease (47.3% and 23.5%, respectively). All patients with cancer (HM or solid tumor with HM) had at least one comorbidity, with immunocompromised being the most common (46.4–69.6%) (Table 1).

Within the sepsis patient population, indications for index events included pneumonia (37.5%), complicated intra-abdominal infection (cIAI) (21.3%), and complicated urinary tract infection (cUTI) (11.6%). In patients with HM, the most common indications for index events were FN (31.3%) and pneumonia (30.4%) while for solid tumor with HM they were cIAI (35.7%) and pneumonia (28.6%). In patients who were immunocompromised and post-transplant, pneumonia (29.5% and 27.5%, respectively) and cUTI (15.7% and 18.8%, respectively) were reported most often. Indications for index events are outlined in Table 2.

Within the sepsis patient population, infections caused by *P. aeruginosa* were the most prevalent (82.5%), of which 61.6% were MDR *P. aeruginosa* cultures. Similarly, 84.1% and 84.4% of infections in immunocompromised and transplant patients, respectively, were attributed to *P. aeruginosa*, of which 71.8% and 71.7% were MDR. In patients with HM, 83.3% had positive cultures for *P. aeruginosa*, 74.2% of which were MDR, and patients with HM with solid tumor displayed 84.2% of *P. aeruginosa* positive cultures, of which 78.9% were MDR. Within the FN patient population, 4.8% of patients were found to have MDR *P. aeruginosa* (Table 2).

### 2.2. Patients with Sepsis

**Figure 1:**
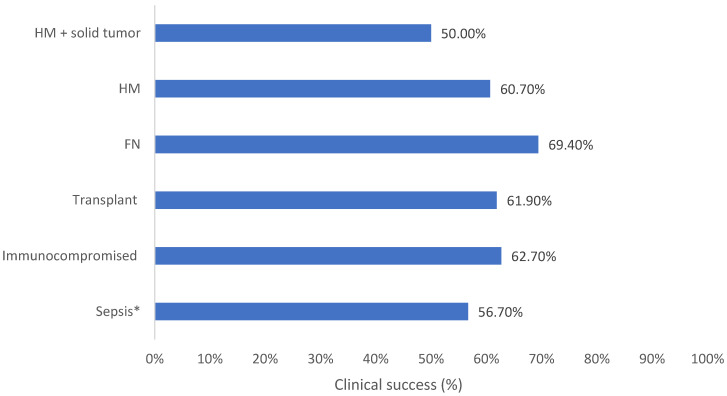
Clinical success for each vulnerable patient population in the SPECTRA study. FN: Febrile neutropenia; HM: Hematologic malignancy. * Sepsis = sepsis/septic shock/systemic inflammatory response syndrome.

Clinical success was observed in 56.7% (95% CI: 50.6–62.6%) of patients with sepsis administered with C/T (Table 3 and Figure 1).

**Figure 2:**
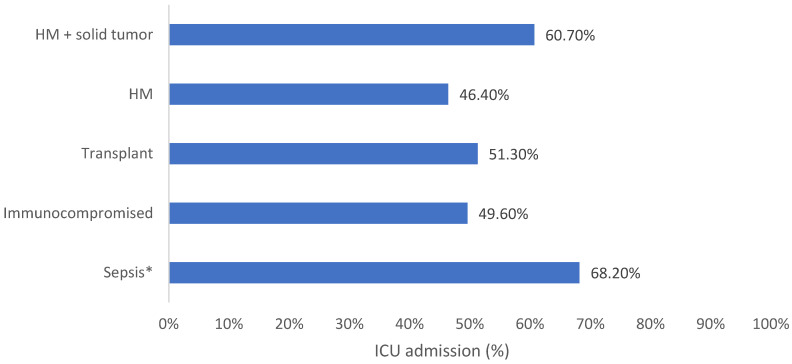
ICU admission for each vulnerable patient population in the SPECTRA study. FN: Febrile neutropenia; HM: Hematologic malignancy; ICU: Intensive care unit. * Sepsis = sepsis/septic shock/systemic inflammatory response syndrome.

**Figure 3:**
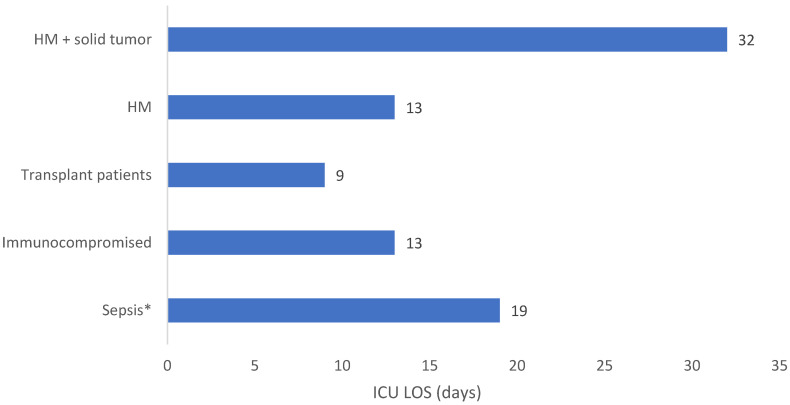
ICU LOS for each vulnerable patient population in the SPECTRA study. FN: Febrile neutropenia; HM: Hematologic malignancy; ICU: Intensive care unit; LOS: Length of stay. * Sepsis = sepsis/septic shock/systemic inflammatory response syndrome.

In this population, 48.3% of patients were admitted to the ICU, among whom 41.9% of ICU admissions were related to the index infection (Table 3 and Figure 2). The median ICU LOS was 19 days within the sepsis population (Table 3 and Figure 3).

**Figure 4:**
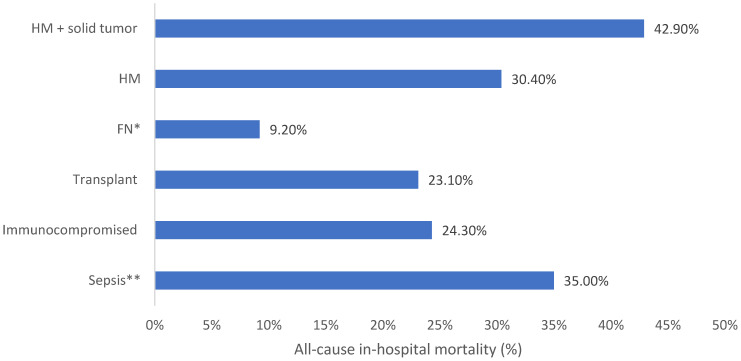
All-cause in-hospital mortality for each vulnerable patient population in the SPECTRA study. FN: Febrile neutropenia; HM: Hematologic malignancy. * Results may be confounded by baseline illness severity. This figure represents the proportion of overall deaths among patients with febrile neutropenia in the available data. When considering mortality within the FN cohort specifically, an elevated cohort mortality rate (31.6%) is seen which likely reflects greater baseline illness severity rather than a direct treatment effect. ** Sepsis = sepsis/septic shock/systemic inflammatory response syndrome.

All-cause in-hospital mortality was reported in 35.0% (95% CI: 29.4–41.0%) of patients with a median time from index date to death of 25 days and a median time from C/T initiation to death of 14 days. All-cause in-hospital mortality in patients in critical care with sepsis was 41.8% (Table 3 and Figure 4).

### 2.3. Immunocompromised and Solid Organ Transplant Patients

Clinical success was reported in 62.7% (95% CI: 56.6–68.5%) of immunocompromised patients and 61.9% (95% CI: 53.9–69.4%) of solid organ transplant patients (Table 3 and Figure 1).

In this population, 49.6% and 51.3% of patients were admitted to the ICU, of which 45.9% and 48.8% were related to index infection, respectively (Table 3 and Figure 2). The median ICU LOS was 13 days for immunocompromised patients and 9 days for solid organ transplant patients (Table 3 and Figure 3).

All-cause in-hospital mortality was similar between immunocompromised and solid organ transplant patients (24.3%, 95% CI: 19.2–29.8% and 23.1%, 95% CI: 16.8–30.4%, respectively). Median time to death from the index date was 29 and 35 days, respectively (Table 3 and Figure 4). The leading causes of death for immunocompromised and solid organ transplant patients were septic shock (12.3%) and multiorgan failure (15.4%).

### 2.4. Febrile Neutropenia

Clinical success was achieved in 69.4% of patients with FN, with a median treatment duration of 8 days (Table 3 and Figure 1). In patients with FN, the proportion of overall deaths within the available data was 9.2% (Table 3 and Figure 4). Within the FN cohort specifically, mortality was 31.6%; however, this result may be confounded by baseline illness severity and therefore reflect greater baseline illness severity rather than a direct treatment effect.

For FN, key ICU metrics and time-to-death intervals were not reported in the available abstraction summaries and therefore cannot be presented.

### 2.5. Cancer (Hematological Malignancy/Solid Tumor)

In the population with cancer, 60.7% (95% CI: 51.0–69.8%) of patients with HM and 50.0% (95% CI: 30.6–69.4%) of patients with HM with solid tumor achieved clinical success (Table 3 and Figure 1).

ICU admission was reported in 46.4% of HM patients and 60.7% and HM with solid tumor patients with a median ICU LOS of 32 days and 13 days, respectively (Table 3, Figure 2 and Figure 3).

All-cause in-hospital mortality was observed in 30.4% (95% CI: 22.0–39.8%) of HM patients and in 42.9% (95% CI: 24.5–62.8%) of HM with solid tumor patients. Time to death from the index date was 31 days and 26 days, respectively (Table 3 and Figure 4).

## 3. Discussion

The SPECTRA study suggests that C/T may be effective in managing severe infections in vulnerable patient populations such as sepsis patients, immunocompromised and transplant patients, cancer patients, and patients with FN. The observed clinical success rates and relatively low mortality rates underscore the potential of C/T as a treatment of MDR infections, which may lead to reduced HCRU.

The sub-analysis of patients with sepsis revealed a clinical success rate of 56.7% and an all-cause in-hospital mortality rate of 35.0%. Similar findings were reported in CACTUS, a multicenter, retrospective, observational, real-world study of 420 patients with *P. aeruginosa* infections. Severe sepsis and septic shock were reported in 59% of the CACTUS patient population, and 62.3% receiving C/T were immunocompromised, with similar baseline patient characteristics to our study. The CACTUS study reported clinical success in 61% of the C/T administered group and a 30-day mortality of 23%, which aligns with the findings of this study. The high rate of ICU admissions (48.3%) and the median ICU LOS (19.0 days) within the sepsis population reflect the significant resource utilization required to manage these patients. The CACTUS study also reported an ICU LOS of 14.7 days [18]. The findings of the CACTUS study are similar, supporting the validity of SPECTRA and the applicability of C/T in real-world settings.

ICU admissions and ICU LOS ranged from 46.4 to 68.2% and 9–32 days, respectively, across all noted vulnerable patient populations, except in patients with FN where ICU admission data was not available. The lowest ICU admission rates were observed within the HM population, while the lowest ICU LOS was reported in transplant patients. The median ICU LOS was 13 days for immunocompromised patients and 9 days for transplant patients, while ICU LOS for patients with HM and HM with solid tumor were 32 and 13 days, respectively. Extended LOS is a contributor to increased HCRU and associated costs. Vulnerable patient populations may already contribute to increased HCRU, particularly those with chronic conditions like immunocompromised states [6,9]. Therefore, appropriate treatment for AMR infections in these vulnerable populations is critical both to ensure optimal care and improve patient outcomes and reduced burden to healthcare systems.

The high prevalence of infections caused by MDR *P. aeruginosa* in our study (61.6% within the sepsis population, 71.8% and 71.7% in immunocompromised and transplant patients and 74.2% and 78.9% for HM and HM with solid tumor, respectively) highlights the critical need for effective treatment options. A US real-world study of 199 patients with *P. aeruginosa* treated with C/T reported 54% ICU admission with a LOS of 18 days, which is comparable to the overall findings of this study [19].

The data suggests that C/T may be beneficial for infections within vulnerable patient populations, particularly those caused by MDR *P. aeruginosa*. The clinical success rate was highest within the FN patient population. However, this population included only 38 patients, which limits the ability to extrapolate conclusions. All-cause in-hospital mortality was lowest in the solid organ transplant population. ICU admission was lowest within the HM population; however, the lowest ICU LOS was observed in transplant patients.

This analysis of vulnerable patient populations provides information which may be important in clinical decision making to improve morbidity and mortality rates and reduce HCRU burden on healthcare systems globally. The data also emphasizes the importance of antimicrobial stewardship and the need for ongoing research to optimize treatment protocols. This is especially important in vulnerable patient populations as AMR has a greater impact on immunocompromised individuals due to prolonged treatment and frequent hospital visits [20].

## 4. Materials and Methods

Study of Prescribing patterns and Effectiveness of Ceftolozane/Tazobactam Real-world Analysis (SPECTRA) was a multicenter, observational study conducted across seven countries (Australia, Austria, Germany, Italy, Spain, Mexico, and the United Kingdom) between 2016 and 2020 which aimed to describe C/T use in hospitalized adults.

### 4.1. Study Design

Patients aged 18 years or older, who received at least 48 h of C/T and received the last dose of C/T ≥ 30 days before data chart abstraction, were included. Patients were excluded from the study if they participated in an interventional Gram-negative infection clinical trial at the time of C/T administration.

The primary objective was to characterize real-world utilization and treatment patterns, clinical outcomes, and healthcare resource utilization associated with C/T in hospitalized adults who received ≥48 h of therapy. A key secondary endpoint was analysis by subpopulations, including within vulnerable populations. The study protocol and statistical analysis plan prespecified a descriptive analytic approach and recognized subgroup analyses as exploratory and conditional on available sample sizes. A comprehensive methodology is reported elsewhere [21].

Per the prespecified protocol, several vulnerable patient populations were defined and are reported in this manuscript. Patients with sepsis as reported in this manuscript had clinical documentation of sepsis, septic shock, or systemic inflammatory response syndrome attributable to the index Gram-negative infection.

Patients classified as immunocompromised included patients with cancer and patients with a documented history of solid organ transplantation. Patients with cancer as reported in this manuscript had HM or HM and solid tumor and were receiving immunosuppressive therapy or chemotherapy. Cancer is presented as a combined category in the summary text; however, where informative, HM and HM with solid tumor are shown as disaggregated counts in detailed tables. Readers should consult table footnotes for which presentation is used. Similarly, solid organ transplant patients are included within the Immunocompromised category but are listed as a subcategory for descriptive clarity.

Patients with FN formed a subcategory of those patients with cancer who were immunocompromised. Patients with FN as reported in this manuscript had documentation of neutropenia with fever temporally associated with the index infection. As FN was reported as a subgroup of cancer patients, separate patient-level characteristics for FN were not presented independently. However, descriptive and exploratory analysis of patients with FN are reported in the detailed tables.

Vulnerable patient groups were not mutually exclusive and, where appropriate, patients were included under more than one vulnerable group definition. For transparency we present counts for each group in the detailed tables and use explicit denominators and footnotes to indicate available-data denominators and non-mutual exclusivity.

This study was conducted in accordance with the International Society for Pharmacoepidemiology Guidelines for Good Pharmacoepidemiology Practices, the ethical principles arising from the Declaration of Helsinki, the European Union good pharmacovigilance practices, European and national laws in terms of data protection, and all applicable local regulations [22,23].

### 4.2. Measurements and Outcomes

Data were retrospectively extracted from medical records for up to 30 days post treatment or until death. Outcomes assessed included clinical success, all-cause in-hospital mortality, ICU admission, and ICU length of stay (LOS). Clinical success was defined as microbiological eradication, no Gram-negative therapy needed for a minimum of 48 h after administration of C/T (not including discharge antibiotics or de-escalation), as well as no death due to Gram-negative infection, no additional treatment for exacerbation of respiratory infection within ≤28 days of stopping C/T, resolution of any exacerbations, or no need for re-operation for source control. Microbiological eradication was defined as negative culture at the site of index infection after administration of C/T. Outcomes reporting on the full included patient population are reported elsewhere [21].

### 4.3. Statistical Analysis

Analyses in this study were descriptive. Percentages were calculated using available-data denominators (number of patients with non-missing data for the variable) and numerators and denominators. For each table and figure, we explicitly report the numerator and denominator used and the number of patients with missing/unknown data. Where data were insufficient for reliable estimation or were suppressed for privacy (small cell counts), we indicate the reason in the table footnote. Continuous variables were presented as mean (standard deviation) and median (interquartile range) where appropriate. Categorical variables were presented as counts and percentages with exact binomial (Clopper–Pearson) 95% confidence intervals for key proportions. Subgroup comparisons were exploratory and were not adjusted for potential confounders as the study was not designed to support causal inference. These analytic principles follow the prespecified statistical analysis plan per protocol, reported elsewhere [21].

## 5. Limitations

The main limitation of this study is the retrospective study design, as it relies on accurate record keeping. Furthermore, bias can be introduced throughout the study. This can include selection bias of patients or sites or possible misclassification bias. Therefore, care should be taken during the interpretation of the results. Furthermore, the lack of comparator or control arm in this study reduces the ability to compare outcomes against the standard of care, thus limiting relative efficacy assessments. Additionally, sample sizes from each country for each patient population vary, and a limited number of patients were included in the FN or solid tumor and HM subgroups. Moreover, subgroups were not mutually exclusive and therefore additional comorbidities may have impacted findings for other groups where patients were included in more than one subgroup. Baseline illness severity scores were not available for FN patients, possibly confounding results and resulting in an inflated mortality rate. ICU data was not available for all subgroups, meaning no conclusions can be drawn on HCRU for patients with FN.

## 6. Conclusions

The data suggest that C/T can play a crucial role in managing complex infections, particularly those caused by MDR *P. aeruginosa*, when integrated into a broader antimicrobial stewardship strategy. SPECTRA suggests that C/T may be effective in managing severe infections in vulnerable patient populations, including those with sepsis, FN, cancer, and immunocompromised or transplant patients. The clinical success rates observed across these subgroups underscore the potential of C/T as a critical component in the treatment of MDR infections. The findings from SPECTRA emphasize the importance of antimicrobial stewardship in managing severe infections in vulnerable populations. These findings supports the use of C/T as a promising therapeutic option for treating infections caused by MDR strains. Continued exploration of combination therapies and optimal treatment protocols will be essential to further enhance outcomes in these vulnerable populations.

Further research is warranted to explore the long-term outcomes of C/T treatment in these patient populations. Studies focusing on combination therapies, dosing regimens, and the impact of antimicrobial stewardship interventions will provide valuable insights into optimizing the use of C/T. Additionally, real-world evidence from diverse healthcare settings will help generalize the findings and support the broader adoption of C/T in clinical practice.

## Figures and Tables

**Table 1:** Patient characteristics for each vulnerable patient population *.

Baseline Characteristics	Sepsis	Immunocompromised	Solid Organ Transplant	HM	Solid Tumor + HM
**Age (years)**					
Mean (SD)	57.4 (16.2)	54.2 (16.4)	50.3 (16.3)	56.3 (15.8)	63.8 (10.6)
Median (Q1; Q3)	59 (47.0; 70.0)	57 (43.0; 67.0)	50.5 (36.5; 64.0)	58 (47.0; 68.0)	62 (56.0; 73.5)
**Gender**					
Male	183/277 (66.1%)	177/268 (66.0%)	108/160 (67.5%)	77/112 (68.8%)	21/28 (75.0%)
Female	94/277 (33.9%)	91/268 (34.0%)	52/160 (32.5%)	35/112 (31.3%)	7/28 (25.0%)
**BMI (kg/m^2^)**					
Mean (SD)	26.1 (6.8)	24.4 (5.8)	24.6 (6.5)	24.5 (4.7)	24.8 (4.7)
Median (Q1; Q3)	25.2 (22.2; 28.3)	24.2 (20.6; 27.0)	23.9 (20.2; 27.3)	24.5 (21.6; 27.0)	24.9 (21.5; 28.0)
**Previous care setting**					
Home/Community	221/277 (79.8%)	218/268 (81.3%)	122/160 (76.3%)	102/112 (91.1%)	27/28 (96.4%)
Other hospital	35/277 (12.6%)	29/268 (10.8%)	20/160 (12.5%)	6/112 (5.4%)	1/28 (3.6%)
Other skilled care facility	16/277 (5.8%)	14/268 (5.2%)	12/160 (7.5%)	3/112 (2.7%)	0/28
Unknown	5/277 (1.8%)	7/268 (2.6%)	6/160 (3.8%)	1/112 (0.9%)	0/28
**At least one comorbidity**	220/277 (79.4%)	268/268 (100%)	160/160 (100%)	112/112 (100%)	28/28 (100%)
**Number of comorbidities (mean)**					
Heart Disease	65/277 (23.5%)	61/268 (22.8%)	37/160 (23.1%)	19/112 (17.0%)	7/28 (25.0%)
Chronic Kidney Disease	50/277 (18.1%)	71/268 (26.5%)	60/160 (37.5%)	15/112 (13.4%)	6/28 (21.4%)
Chronic Pulmonary Disease	64/277 (23.1%)	75/268 (28.0%)	46/160 (28.8%)	15/112 (13.4%)	5/28 (17.9%)
Cystic Fibrosis	15/277 (5.4%)	36/268 (13.4%)	33/160 (20.6%)	0/112	0/28
Liver Disease (moderate to severe)	17/277 (6.1%)	17/268 (6.3%)	10/160 (6.3%)	7/112 (6.3%)	1/28 (3.6%)
Diabetes Mellitus (end organ damage)	13/277 (4.7%)	22/268 (8.2%)	19/160 (11.9%)	3/112 (2.7%)	1/28 (3.6%)
Immunocompromised	101/277 (36.5%)	192/268 (71.6%)	112/160 (70.0%)	78/112 (69.6%)	13/28 (46.4%)
HM	65/277 (23.5%)	112/268 (41.8%)	41/160 (25.6%)	112/112 (100%)	28/28 (100%)
Transplant	74/277 (26.7%)	160/268 (59.7%)	160/160 (100%)	41/112 (36.6%)	4/28 (14.3%)

Units are expressed as *n* (%) unless stated otherwise. * Due to small sample size (*n* = 38), baseline information for patients with FN was not reported separately in this study. Please note, percentages use available-data denominators (numerator/denominator shown where applicable). Cancer is reported as a combined category in summary text; disaggregated HM counts are detailed in tables where available. Solid organ transplant patients are included within the Immunocompromised group and are shown separately for descriptive clarity. ‘NR’ indicates the value was not reported in the source materials. HM: Hematologic malignancy; SD: Standard deviation; SIRS: Systemic inflammatory response syndrome; Q: Quartile.

**Table 2:** Indication for index event and MDR organisms for each vulnerable patient population.

	Sepsis	Immunocompromised	Solid Organ Transplant	FN	HM	Solid Tumor + HM
**Indication for index event**						
cIAI	59/277 (21.3%)	38/268 (14.2%)	20/160 (12.5%)	1/38 (2.6%)	13/112 (11.6%)	10/28 (35.7%)
cUTI	32/277 (11.6%)	42/268 (15.7%)	30/160 (18.8%)	2/38 (5.3%)	13/112 (11.6%)	4/28 (14.3%)
Pneumonia	104/277 (37.5%)	79/268 (29.5%)	44/160 (27.5%)	3/38 (7.9%)	34/112 (30.4%)	8/28 (28.6%)
Exacerbation of CRI	18/277 (6.5%)	28/268 (10.4%)	19/160 (11.9%)	NR	4/112 (3.6%)	1/28 (3.6%)
Sepsis	113/277 (40.8%)	61/268 (22.8%)	39/160 (24.4%)	4/38 (10.5%)	24/112 (21.4%)	6/28 (21.4%)
FN	22/277 (7.9%)	37/268 (13.8%)	17/160 (10.6%)	NR	35/112 (31.3%)	4/28 (14.3%)
Bone and Joint Infection	7/277 (2.5%)	8/268 (3.0%)	8/160 (5.0%)	NR	1/112 (0.9%)	0/28
Prophylaxis	0/277	3/268 (1.1%)	3/160 (1.9%)	NR	0/112	0/28
Bacteremia	8/277 (2.9%)	7/268 (2.6%)	4/160 (2.5%)	NR	5/112 (4.5%)	2/28 (7.1%)
Skin	7/277 (2.5%)	10/268 (3.7%)	4/160 (2.5%)	1/38 (2.6%)	5/112 (4.5%)	1/28 (3.6%)
Respiratory	5/277 (1.8%)	9/268 (3.4%)	5/160 (3.1%)	4/38 (10.5%)	4/112 (3.6%)	3/28 (10.7%)
Wound	8/277 (2.9%)	10/268 (3.7%)	6/160 (3.8%)	NR	6/112 (5.4%)	1/28 (3.6%)
Other Indication	6/277 (2.2%)	7/268 (2.6%)	7/160 (4.4%)	NR	3/112 (2.7%)	0/28
Unknown	0/277	1/268 (0.4%)	1/160 (0.6%)	NR	0/112	0/28
Not Applicable	3/277 (1.1%)	10/268 (3.7%)	4/160 (2.5%)	NR	5/112 (4.5%)	0/28
**MDR organisms**						
MDR *P. aeruginosa*	109 (61.6%)	122 (71.8%)	71 (71.7%)	15 (39.5%)	49 (74.2%)	15 (78.9%)
MDR Enterbacterecea	16 (9.0%)	12 (7.1%)	7 (7.1%)	NR	2 (10.5%)	16 (9.0%)

Note: Percentages use available-data denominators (numerator/denominator shown where applicable). Cancer is reported as a combined category in summary text; disaggregated HM counts are detailed in tables where available. Solid organ transplant patients are included within the Immunocompromised group and are shown separately for descriptive clarity. ‘NR’ indicates the value was not reported in the source materials. CI: Confidence interval; cIAI: Complicated intra-abdominal infection; cUTI: Complicated urinary tract infection; CRI: Chronic respiratory infection; FN: Febrile neutropenia; HM: Hematologic malignancy; MDR: Multi-drug resistant: NR: Not reported; SD: Standard deviation.

**Table 3:** Clinical and HCRU outcomes for each vulnerable patient population.

	Sepsis	Immunocompromised	Solid Organ Transplant	FN *	HM	HM and Solid Tumor
**Index infection considered as a clinical success by the investigator**						
*n* (%)	157/277 (56.7%)	168/268 (62.7%)	99/160 (61.9%)	25/38 (69.4%)	68/112 (60.7%)	14/28 (50.0%)
95% CI	50.6%, 62.6%	56.6%, 68.5%	53.9%, 69.4%	52.9–83.0	51.0%, 69.8%	30.6%, 69.4%
**All-cause in-hospital mortality**						
*n* (%)	97/277 (35.0%)	65/268 (24.3%)	37/160 (23.1%)	12/131 (9.2%) **	34/112 (30.4%)	12/28 (42.9%)
95% CI	29.4%, 41.0%	19.2%, 29.8%	16.8%, 30.4%	NR	22.0%, 39.8%	24.5%, 62.8%
**Admission in ICU during the index hospitalization**						
*n* (%)	189/277 (68.2%)	133/268 (49.6%)	82/160 (51.3%)	NR	52/112 (46.4%)	17/28 (60.7%)
**ICU related to index infection**						
*n* (%)	95/277 (50.3%)	61/268 (45.9%)	40/160 (48.8%)	NR	24/112 (46.2%)	8/28 (47.1%)
**If ICU related to index infection, ICU length of stay (days)**						
Mean (SD)	23.4 (19.6)	21.9 (22.9)	20.7 (22.3)	NR	20.4 (18.8)	34.0 (26.3)
95% CI	(19.4, 27.4)	(16.0, 27.8)	(13.5, 27.9)	NR	(12.4, 28.3)	(12.0, 56.0)
Median (Q1; Q3)	19 (8.0; 36.0)	13 (6.5; 29.0)	9 (5.0; 36.0)	NR	13 (8.0; 26.0)	32 (11.5; 50.5)
**Time from index date to death (days)**						
Mean (SD)	33.9 (29.4)	34.8 (25.3)	34.8 (21.8)	NR	35.2 (25.6)	38.3 (34.2)
Median (Q1; Q3)	25 (15.0; 47.0)	29 (15.0; 47.0)	35 (15.0; 48.0)	NR	31 (18.0; 44.0)	25.5 (14.0; 46.5)
**Time from C/T initiation to death (days)**						
Mean (SD)	22.0 (27.3)	23.3 (22.8)	21.6 (18.2)	NR	23.5 (23.5)	33.5 (34.2)
Median (Q1; Q3)	14 (6.0; 25.0)	16 (7.0; 33.0)	15 (7.0; 33.0)	NR	17 (9.0; 33.0)	23.5 (10.0; 46.5)

Units are expressed as *n* (%) unless stated otherwise. Cancer is reported as a combined category in summary text; disaggregated HM counts are detailed in tables where available. Solid organ transplant patients are included within the Immunocompromised group and are shown separately for descriptive clarity. ‘NR’ indicates the value was not reported in the source materials. Where exact denominators were available, exact binomial 95% CIs were calculated. ‘NR’ indicates the CI was not reported in the source materials because a denominator for that metric was not available. * For FN, two patients had missing clinical success status. Additionally, key ICU metrics and time-to-death intervals were not reported in the available abstraction summaries and therefore cannot be presented. ** Results may be confounded by baseline illness severity. This figure represents the proportion of overall deaths among patients with febrile neutropenia in the available data. When considering mortality within the FN cohort specifically, an elevated cohort mortality rate (31.6%) is seen, which likely reflects greater baseline illness severity rather than a direct treatment effect. CI: Confidence interval: FN: Febrile neutropenia; HM: Hematologic malignancy; *n*: Number; SD: Standard deviation; SIRS: Systemic inflammatory response syndrome; Q: Quartile.

## Data Availability

The full datasets generated and analyzed to inform the conclusions drawn within this manuscript during the current study are available from the corresponding author upon reasonable request.
